# Supplementary material for: Molecular surveillance of hepatitis E virus in wastewater in Yaoundé, Cameroon
Source: PLoS One. 2025 Aug 13;20(8):e0322765. doi: 10.1371/journal.pone.0322765 (PMC12348994; doi:10.1371/journal.pone.0322765)
Supplement: S2 Table — Summarize Result of HEV from wastewater. (PDF) [file pone.0322765.s002.pdf]

Sample sit	month of collect	Extraction kit	Resultats gel	Intensity of the signal on UV	Sequence obtained after sequecing	Access number	Sequence
**BIYEM 01**	January	DaAnGene	Negative				
**BIYEM 02**	February	DaAnGene	Detected with non-specific amplification	+++	No		
**BIYEM 03**	March	DaAnGene	Detected with non-specific amplification	+	No		
**BIYEM 04**	April	DaAnGene	Detected	+++	Yes	PP764563	TGTTTGGTCCGTGGTTTGGGGCCATTGAGAAGGCAATT GTGGATGTGCTGCCTGAGTGGTGCTTTTATGGCGATTG TTATGTGCAGGAAAAACTGGAGGCCGCTGTGGCGGGG GCCGAGGCGTGCCGGGTTTTTGAGAATGACTTTGGTGA GTTTGATAGCACGCAGAATAATTATTCCCTGGGCCTGG AGTGCCTATTGTTGCAGGAAGCTGGGGCCCCTGAATGG CTATGGAGACTATATCATCTGCTCCGCTCGGCGTGGGT GCTACAAGCCCCCCAGGAGAGTCTGCGAGGCCGGTGG AAGAAACACCCCGGCGAACCCG
**BIYEM 05**	May	DaAnGene	Negative				
**BIYEM 06**	June	DaAnGene	Negative				
**BIYEM 07**	July	DaAnGene	Negative				
**BIYEM 08**	August	DaAnGene	Negative				
**BIYEM 09**	September	DaAnGene	Negative				
**BIYEM 10**	October	DaAnGene	Negative				
**BIYEM 11**	November	DaAnGene	Negative				
**BIYEM 12**	December	DaAnGene	Negative				
**BONAS 01**	January	DaAnGene	Negative				
**BONAS 02**	February	DaAnGene	Detected with non-specific amplification	+	No		

**BONAS 03**	March	DaAnGene	Negative				
**BONAS 04**	April	DaAnGene	Negative				
**BONAS 05**	May	DaAnGene	Negative				
**BONAS 06**	June	DaAnGene	Detected	+++	Yes	PP764566	TGTTTGGTCCGTGGTTTGGGGCCATTGAGAAGGCAATT<br>GTGGATGTGCTGCCTGAGTGGTGCTTTTATGGCGATTG<br>TTATGTGCAGGAAAAACTGGAGGCCGCTGTGGCGGGG<br>GCCGAGGCGTGCCGGGTTTTTGAGAATGACTTTGGTGA<br>GTTTGATAGCACGCAGAATAATTATTCCCTGGGCCTGG<br>AGTGCCTATTGTTGCAGGAAGCTGGGGCCCCTGAATGG<br>CTATGGAGACTATATCATCTGCTCCGCTCGGCGTGGGT<br>GCTACAAGCCCCCCAGGAGAGTCTGCGAGGCCGGTGG<br>AAGAAACACCCCGGCGAACCCG
**BONAS 07**	July	DaAnGene	Detected with non-specific amplification	+	No		
**BONAS 08**	August	DaAnGene	Negative				
**BONAS 09**	September	DaAnGene	Negative				
**BONAS 10**	October	DaAnGene	Detected	+++	Yes	PP764564	TGTTTGGTCCGTGGTTTGGGGCCATTGAGAAGGCAATT<br>GTGGATGCGCTGCCCGAGTGGTGCTTTTATGGAGACTG<br>TTATGTACAAGAAAAACTGGAGGCTGCCGTGGCGGGG<br>GCTGAGGCGTGCCGGGTTTTCGAGAATGATTTTGGTGA<br>GTTTGACAGCACGCAGAATAACTATTCCTTGGGCCTGG<br>AGTGTCTCTTGTTGCAGGAAGCTGGGGCCCCTGAATGG<br>TTGTGGAGATTATATCATCTGCTCCGCTCGGCGTGGGT<br>GTTGCAGGCCCCCCAAGAGAGTTTGCGAGGTCGGTGG<br>AAGAAACACCCCGGCGAACCCG

**BONAS 11**	November	DaAnGene	Detected	+++	Yes	PP764565	TGTTTGGTCCGTGGTTTGGGGCCATTGAGAAGGCAATTGTGGATGCATTGCCCGAATGGTGCTTTTACGGAGACTGTTATGTACAAGAAAAGCTGGAGGCCGCCGTGGCGGGGGCCGAGGCGTGCCGGGTCTTTGAGAATGATTTTGGTGAGTTCGACAGTACGCAGAATAACTATTCCCTGGGTTTGGAGTGTCTGTTGTTGCAGGAGGCTGGGGCCCCTGAATGGCTGTGGAGGTTGTATCATCTGCTCCGCTCGGCGTGGGTGTTGCAGGCCCCCCAAGAGAGTTTGCGGGGTCGGTGGAAGAAACACCCCGGCGAACCCG
**BONAS 12**	December	DaAnGene	Negative				
**CHUY 01**	January	DaAnGene	Negative				
**CHUY 02**	February	DaAnGene	Negative				
**CHUY 03**	March	DaAnGene	Detected with non-specific amplification	+	No		
**CHUY 04**	April	DaAnGene	Negative				
**CHUY 05**	May	DaAnGene	Negative				
**CHUY 06**	June	DaAnGene	Negative				
**CHUY 07**	July	DaAnGene	Negative				
**CHUY 08**	August	DaAnGene	Negative				
**CHUY 09**	September	DaAnGene	Negative				
**CHUY 10**	October	DaAnGene	Negative				
**CHUY 11**	November	DaAnGene	Negative				
**CHUY 12**	December	DaAnGene	Negative				
**DIDEROT 01**	January	DaAnGene	Negative				
**DIDEROT 02**	February	DaAnGene	Negative				
**DIDEROT 03**	March	DaAnGene	Negative				
**DIDEROT 04**	April	DaAnGene	Negative				
**DIDEROT 05**	May	DaAnGene	Negative				
**DIDEROT 06**	June	DaAnGene	Detected with smears	+++	No		
**DIDEROT 07**	July	DaAnGene	Negative				

**DIDEROT 08**	August	DaAnGene	Negative				
**DIDEROT 09**	September	DaAnGene	Detected	+++	Yes	PP764568	TGTTTGGTCCGTGGTTTGGGGCCATTGAGAAGGCAATT<br>GTGGATGTGCTGCCTGAGTGGTGCTTTTATGGCGATTG<br>TTATGTGCAGGAAAAACTGGAGGCCGCTGTGGCGGGG<br>GCCGAGGCGTGCCGGGTTTTTGAGAATGACTTTGGTGA<br>GTTTGATAGCACGCAGAATAATTATTCCCTGGGCCTGG<br>AGTGCCTATTGTTGCAGGAAGCTGGGGCCCCTGAATGG<br>CTATGGAGACTATATCATCTGCTCCGCTCGGCGTGGGT<br>GCTACAAGCCCCCCAGGAGAGTCTGCGCGGCCGGTGG<br>AAGAAACACCCCGGCGAACCCG
**DIDEROT 10**	October	DaAnGene	Negative				
**DIDEROT 11**	November	DaAnGene	Detected	+++	Yes	PP764567	TGTTTGGTCCGTGGTTTGGGGCCATTGAGAAGGCAATT<br>GTGGATGCGCTGCCCGAGTGGTGCTTTTATGGAGACTG<br>TTATGTACAAGAAAAGCTGGAGGCCGCCGTGGCGGGG<br>GCTGAGGCGTGCCGGGTTTTCGAGAATGATTTTGGCGA<br>GTTTGACAGCACGCAGAACAACTATTCCTTGGGCCTGG<br>AGTGTCTCTTGTTGCGGGAAGCTGGGGCCCCTGAATGG<br>TTGTGGAGATTATATCATCTGCTCCGCTCGGCGTGGGT<br>GTTGCAGGCCCCCCAAGAGAGTTTGCGGGGCCGGTGG<br>AAGAAACACCCCGGCGAACCCG
**DIDEROT 12**	December	DaAnGene	Negative				
**MVOGA 01**	January	DaAnGene	Negative				
**MVOGA 02**	February	DaAnGene	Negative				
**MVOGA 03**	March	DaAnGene	Negative				
**MVOGA 04**	April	DaAnGene	Negative				
**MVOGA 05**	May	DaAnGene	Negative				
**MVOGA 06**	June	DaAnGene	Detected with non-specific amplification	++	No		
**MVOGA 07**	July	DaAnGene	Negative				

**MVOGA 08**	August	DaAnGene	Detected with smears	+++	No		
**MVOGA 09**	September	DaAnGene	Negative				
**MVOGA 10**	October	DaAnGene	Negative				
**MVOGA 11**	November	DaAnGene	Negative				
**MVOGA 12**	December	DaAnGene	Negative				
**PAPOSY 01**	January	DaAnGene	Negative				
**PAPOSY 02**	February	DaAnGene	Negative				
**PAPOSY 03**	March	DaAnGene	Negative				
**PAPOSY 04**	April	DaAnGene	Detected with non-specific amplification	++	No		
**PAPOSY 05**	May	DaAnGene	Negative				
**PAPOSY 06**	June	DaAnGene	Negative				
**PAPOSY 07**	July	DaAnGene	Detected with non-specific amplification	++	No		
**PAPOSY 08**	August	DaAnGene	Detected with non-specific amplification	++	No		
**PAPOSY 09**	September	DaAnGene	Detected	+++	Yes	PP764569	TGTTTGGTCCGTGGTTTGGGGCCATTGAGAAGGCAATT<br>GTGGATGCGCTGCCCGAGTGGTGCTTTTATGGAGACTG<br>TTATGTACAAGAAAAGCTGGAGGCTGCCGTGGCGGGG<br>GCTGAGGCGTGTCGGGTTTTCGAGAATGATTTCGGCGA<br>GTTTGACAGCACGCAGAACAACTATTCCTTGGGCCTGG<br>AGTGCCTCTTGTTGCAGGAGGCTGGGGCCCCTGAATG<br>GTTGTGGAGATTATATCATCTGCTCCGCTCGGCGTGGG<br>TGTTGCAGGCCCCCCAAGAGAGTTTGCGGGGCCGGTG<br>GAAGAAACACCCCGGCGAACCCG
**PAPOSY 10**	October	DaAnGene	Detected with non-specific amplification	++	No		

**PAPOSY 11**	November	DaAnGene	Negative				
**PAPOSY 12**	December	DaAnGene	Negative				
