# Supplementary material for: Molecular surveillance of hepatitis E virus in wastewater in Yaoundé, Cameroon
Source: PLoS One. 2025 Aug 13;20(8):e0322765. doi: 10.1371/journal.pone.0322765 (PMC12348994; doi:10.1371/journal.pone.0322765)
Supplement: Attachment — Submitted filename: Response to reviewers.docx [file pone.0322765.s004.docx]

Journal requirements:

When submitting your revision, we need you to address these additional requirements.

1. Please ensure that your manuscript meets PLOS ONE's style requirements, including those for file naming. The PLOS ONE style templates can be found at https://journals.plos.org/plosone/s/file?id=wjVg/PLOSOne_formatting_sample_main_body.pdf and

[https://journals.plos.org/plosone/s/file?id=ba62/PLOSOne_formatting_sample_title_authors_affiliations.pdf](https://track.editorialmanager.com/CL0/https:%2F%2Fjournals.plos.org%2Fplosone%2Fs%2Ffile%3Fid=ba62%2FPLOSOne_formatting_sample_title_authors_affiliations.pdf/1/010f0196ce6a3257-83474c83-0763-4b86-8e80-b7d515349186-000000/RP4XoxOogvwAeJcwoAgGpApZNWsbN92M4QzAh7K1-UI=211)

Thank you for the reminder. We have updated the manuscript to fully comply with PLOS ONE’s style requirements.

2. Please include a complete copy of PLOS’ questionnaire on inclusivity in global research in your revised manuscript. Our policy for research in this area aims to improve transparency in the reporting of research performed outside of researchers’ own country or community. The policy applies to researchers who have travelled to a different country to conduct research, research with Indigenous populations or their lands, and research on cultural artefacts. The questionnaire can also be requested at the journal’s discretion for any other submissions, even if these conditions are not met.  Please find more information on the policy and a link to download a blank copy of the questionnaire here: https://journals.plos.org/plosone/s/best-practices-in-research-reporting. Please upload a completed version of your questionnaire as Supporting Information when you resubmit your manuscript.”

In this study, the PLOS questionnaire on inclusivity in global research was not used, as the research did not involve travel to a different country, engagement with Indigenous populations or their lands, or work with cultural artefacts. All research activities were conducted within the researchers’ own country and community.

Thank you for your comments regarding the inclusivity of global research. We appreciate the opportunity to provide more information. The completed questionnaire provides detailed information on our research practices and considerations has been included as a supplementary file in our revised manuscript. We have also completed method section **lines 105-107** as follows: “Further information on scientific considerations relating to inclusivity in global research can be found in the Supporting Information. As a national reference laboratory for emerging and re-emerging epidemic preparedness and response, our work focuses on supporting public health efforts. No permits were required to access the field sites.” We would like to clarify the following points: (1) Research context: Our laboratory's role is to support national health efforts, and this research contributes to that goal. (2). Field site access: No permits were required for accessing the field sites, as wastewater samples were collected from public gutters in areas accessible to the general public. (3). Sampling activities: The sampling did not involve restricted or protected sites, and samples were not transported across state lines.

3. In your Methods section, please provide additional information regarding the permits you obtained for the work. Please ensure you have included the full name of the authority that approved the field site access and, if no permits were required, a brief statement explaining why.

To conform to the Guidance of PLOS, we have added these sentence **lines 105 to 107** as follows: As a national reference laboratory for emerging and re-emerging epidemic preparedness and response, our work focuses on supporting public health efforts. No permits were required to access the field sites. In addition, identification of the sample sites has been performed in collaboration with the staff of the Mfoundi Division. We have completed the Acknowledgements section lines x to y as follows: Acknowledgments: We are grateful to the field staff from the Mfoundi Division who participated in identifying the study sites, and to the workers involved in collecting wastewater samples.

4. We noticed you have some minor occurrence of overlapping text with the following previous publication(s), which needs to be addressed: <https://d.docksci.com/first-evidence-of-the-hepatitis-e-virus-in-environmental-waters-in-colombia_59f0f730d64ab2a3b992cac0.html>. In your revision ensure you cite all your sources (including your own works), and quote or rephrase any duplicated text outside the methods section. Further consideration is dependent on these concerns being addressed.

Thank you for bringing the minor overlap in text to our attention. We apologise for the duplication and have revised the manuscript accordingly. Specifically, we have rewritten the affected sections to eliminate redundancy and ensure originality. We have also thoroughly reviewed the manuscript to ensure that all sources, including our own work, are properly cited.

5. Please include your tables as part of your main manuscript and remove the individual files. Please note that supplementary tables (should remain/ be uploaded) as separate "supporting information" files.

Thank you for the guidance. As suggested, the table has been incorporated into the main manuscript on page 5, and the separate file has been removed.

6. We note that your Data Availability Statement is currently as follows: [All relevant data are within the manuscript and its Supporting Information files.] Please confirm at this time whether or not your submission contains all raw data required to replicate the results of your study. Authors must share the “minimal data set” for their submission. PLOS defines the minimal data set to consist of the data required to replicate all study findings reported in the article, as well as related metadata and methods (<https://journals.plos.org/plosone/s/data-availability#loc-minimal-data-set-definition>). For example, authors should submit the following data:

- The values behind the means, standard deviations and other measures reported;

- The values used to build graphs;

- The points extracted from images for analysis.

Authors do not need to submit their entire data set if only a portion of the data was used in the reported study. If your submission does not contain these data, please either upload them as Supporting Information files or deposit them to a stable, public repository and provide us with the relevant URLs, DOIs, or accession numbers. For a list of recommended repositories, please see <https://journals.plos.org/plosone/s/recommended-repositories>. If there are ethical or legal restrictions on sharing a de-identified data set, please explain them in detail (e.g., data contain potentially sensitive information, data are owned by a third-party organization, etc.) and who has imposed them (e.g., an ethics committee). Please also provide contact information for a data access committee, ethics committee, or other institutional body to which data requests may be sent. If data are owned by a third party, please indicate how others may request data access.

Thank you for your query regarding the data availability statement. We will revise it during the review process by including a dataset that compiles all relevant data in the Supporting Information section.

7. When completing the data availability statement of the submission form, you indicated that you will make your data available on acceptance. We strongly recommend all authors decide on a data sharing plan before acceptance, as the process can be lengthy and hold up publication timelines. Please note that, though access restrictions are acceptable now, your entire data will need to be made freely accessible if your manuscript is accepted for publication. This policy applies to all data except where public deposition would breach compliance with the protocol approved by your research ethics board. If you are unable to adhere to our open data policy, please kindly revise your statement to explain your reasoning and we will seek the editor's input on an exemption. Please be assured that, once you have provided your new statement, the assessment of your exemption will not hold up the peer review process.

We apologise for this restriction and will review the data availability statement accordingly. “Data will be made available upon request to the corresponding author”.

8. Please amend your manuscript to include your abstract after the title page.

We apologise, we have already included our abstract after the title page **(Lines 28 to 54**) page 2.

9. We note that Figure 1 in your submission contain [map/satellite] images which may be copyrighted. All PLOS content is published under the Creative Commons Attribution License (CC BY 4.0), which means that the manuscript, images, and Supporting Information files will be freely available online, and any third party is permitted to access, download, copy, distribute, and use these materials in any way, even commercially, with proper attribution. For these reasons, we cannot publish previously copyrighted maps or satellite images created using proprietary data, such as Google software (Google Maps, Street View, and Earth). For more information, see our copyright guidelines: http://journals.plos.org/plosone/s/licenses-and-copyright.

We require you to either (1) present written permission from the copyright holder to publish these figures specifically under the CC BY 4.0 license, or (2) remove the figures from your submission:

Thank you for your note regarding the copyright of Figure 1. We apologize for this oversight. In accordance with the editor’s request, we have removed Figure 1 (the map of our sample collection sites).

10. Please include a copy of Table 1 which you refer to in your text on page 5.

Thank you for the guidance. As requested, table 1 has been incorporated into the main manuscript on **page 7**, and the separate file has been removed.

**Major comments: Molecular Epidemiology of hepatitis E virus in wastewater in Yaoundé, Cameroon: Toward optimal prevention and control of emerging zoonotic pathogens**

λ The current title seems too big. Consider removing “Toward optimal prevention and control of emerging zoonotic pathogens”.

We agree with Reviewer #1’s comment regarding the length of the original title and support their suggestion to remove the phrase 'Towards optimal prevention and control of emerging zoonotic pathogens'. As requested, we have revised the title to: “Molecular surveillance of hepatitis E virus in wastewater in Yaoundé, Cameroon”.

λ A brief introduction of genomic structure of HEV is suggested, since ORF1 gene has been mentioned in the main text. Additionally, the authors should also mention the classification of HEV-3 subtypes.

We agree with Reviewer #1’s comment regarding the brief introduction of the genomic structure of HEV, the ORF1 gene, and the classification of HEV-3 subtypes. As requested and suggested, we have modified **lines 62-74** of the “Introduction” as well as updated some references as follows: “The hepatitis E virus (HEV) is a non-enveloped, single-stranded, positive-sense RNA virus belonging to the Hepeviridae family, and the Paslahepevirus genus. Its genome is approximately 7.2 kilobases (kb) in length and comprises three open reading frames (ORFs). ORF1 encodes non-structural proteins involved in viral replication, ORF2 encodes the viral capsid protein, and ORF3 encodes a small multifunctional protein associated with virus egress and host interaction [9–11]. Among the eight identified HEV genotypes (HEV-1 to HEV-8), four (HEV-1, HEV-2, HEV-3, and HEV-4) are known to infect humans. HEV genotype 3 (HEV-3) is zoonotic and widely distributed among both humans and animals, particularly in developed and transitional countries. The primary animal hosts of HEV-3 include pigs, rabbits, deer, and mongooses. HEV-3 is further subdivided into multiple subtypes (e.g., 3a to 3j) based on phylogenetic differences. Similarly, HEV genotype 4 (HEV-4) infects both humans and pigs. Genotypes 3, 4, and 7 exhibit zoonotic potential. Genotypes 5 and 6 have been identified in wild boars, while genotypes 7 and 8 have recently been isolated from camelids and dromedaries [9,11]”.

λ Statistical analysis has not been described in Material and Methods.

We apologise for the absence of this part. In line with the guidance and as requested, we have added the relevant information on page 5, **lines 142–145**, in the 'Materials and Methods' section, as follows: 'Statistical analysis: Statistical analyses were performed using IBM SPSS Statistics 25.0 software. Qualitative variables were presented as frequencies, and the chi-square test was used to compare proportions, with a significance level set at p values <0.05”.

λ Figure 1: The texts are difficult to read. Please increase the resolution. Thank you for this observation.

We apologize for this oversight. In accordance with the editor’s request, we have removed Figure 1 (the map of our sample collection sites).

λ Table 1: What is the meaning of “positif”?

We apologise for this misunderstanding. Here, 'positive' refers to detection; we have replaced it in lines 142 and 145 as well as in the table as follows:

**lines 152-156**: As shown in Table 1, HEV was detected in 26.4% (19 out of 72) of wastewater samples. Notably, HEV was identified in samples from all six study locations. The highest detection rates were observed in Bonas and Paposy, at 41.7% (05 out of 12 samples each), while the lowest rate was recorded in CHUY at 8.4% (one out of 12 samples). Biyem Assi and Diderot showed detection rates of 25% (03 out of 12 samples), while Mvog Ada's rate was 16.7% (two out of 12 samples).

Table 1: Summary of hepatitis E virus detection at each collection site

| Sites |  | N tested | N detected | % of detection |
| --- | --- | --- | --- | --- |
| Biyem Assi |  | 12 | 3 | 25 |
| Bonas |  | 12 | 5 | 41.7 |
| CHUY |  | 12 | 1 | 8.4 |
| Diderot |  | 12 | 3 | 25 |
| Mvogada |  | 12 | 2 | 16.7 |
| Paposy |  | 12 | 5 | 41.7 |
| Total |  | 72 | 19 | 26.4 |

*N=Total number of samples, %= percentage,*

λ Line 126: This reviewer does not understand “In this study, 52.6% (10/19) of the samples showed a strong signal for HEV-RNA.” How did the authors determine a strong signal for HEV-RNA? Please specify this in more detail.

We apologise for this misunderstanding. To clarify, the following details have been added to **lines 165–170** on page 6: In this study, 52.6% (10/19) of the HEV-detectable samples exhibited a robust signal under UV illumination after electrophoresis, suggesting a higher concentration of HEV RNA. The signal intensity was assessed visually on the gel and scored on a scale from 1 to 3, where 1 indicates the weakest signal and 3 indicates the strongest. Of the samples that tested strongly positive, 30% (3/10) were from BONAS, 10% (1/10) from CHUY, 20% (2/10) from DIDEROT, 30% (3/10) from PAPOSY, and 10% (1/10) from BIYEMASSI.

λ Figure 2 is difficult to comprehend. Percentage (%) should be at least indicated on the Y axis.

Thank you for the remarks, as suggested, the detail of different percentages was added in the figure 2 as follows:

λ What is the subtype of these HEV-3 strains circulating in this region? Furthermore, the authors should conduct sequence analysis of HEV ORF1 fragments identified in this study. For example, the sequence identifies between them.

Thank you for your insightful comments on our manuscript. We appreciate your suggestion regarding subtypes circulating in this region. To address this issue, we have conducted further analysis of the sequences obtained in the present study. Our analysis revealed a high degree of similarity between our sequences and those of rat hepatitis E Orthohepevirus C genotype C1, as shown in Figure 3 below; The update was added on discussion, conclusion of the manuscript section **line 234-283;** and in result and conclusion of abstract **line 40-51.**

λ Figure 3: In the phylogenetic tree, rat HEV-C1 strains are very divergent thus unnecessary to be shown; in contrast, representative HEV genotypes within Paslahepevirus balayani can be included in the phylogenetic analysis. In addition, please indicate the HEV-3 subtypes on the right of the tree for a better understanding (see PMID: 29514938).

We thank the reviewer for this helpful and thoughtful suggestion. While we acknowledge that many Orthohepevirus C1 strains (particularly those from rodents) are indeed highly divergent and can visually reduce the resolution of phylogenetic relationships among Paslahepevirus balayani genotypes, we have chosen to retain a small, curated subset of C1 sequences in the revised tree. This decision is supported by the fact that our Cameroonian sequences show unexpected and consistent proximity to HEV-C1 strains, rather than to classical HEV genotypes (HEV-1 to HEV-4), as demonstrated by both Maximum Likelihood and Neighbor-Joining approaches. The update was added on discussion, conclusion of the manuscript section **line 234-283;** and in result and conclusion of abstract **line 40-51.**

To balance clarity and interpretability: We removed overly divergent HEV-C1 sequences that were not relevant to the specific clustering observed with our sequences. We retained only representative HEV-C1 strains, including sequences from studies reporting zoonotic infections in humans (e.g., European cases described in Sridhar et al., 2018 and PMID: 28695203), which provide an important comparative framework given the potential public health implications of our findings.

We updated the tree to include a broader and more representative selection of HEV genotypes within Paslahepevirus balayani, particularly HEV-3 subtypes (3a, 3c, 3e, 3f, etc.), in accordance with the classification guidelines described in Smith et al., 2016 (PMID: 29514938). HEV-3 subtype labels have been added to the right side of the tree, as suggested, to improve clarity and facilitate subtype-level interpretation. We believe this revised version of the tree maintains scientific rigor while improving visual clarity and relevance. These updates are now reflected in the new Figure 3 and corresponding legend in the manuscript. We have also clarified this rationale in the discussion section.

We sincerely appreciate this comment, which allowed us to refine our phylogenetic analysis and better highlight the unusual clustering of our sequences with HEV-C1 strains. (Figure 3 up)

λ Discussion: As introduced in line 64, HEV-1 has been detected in Cameroon previously. Also, to the knowledge of this reviewer, both HEV-1 and HEV-2 are circulating in the neighbor country Nigeria (PMID: 30352598). The authors may discuss why no HEV-1/2 has been detected in the wastewater in this study.

Thank you for this valuable observation. We acknowledge the prior detection of HEV-1 in Cameroon and the circulation of both HEV-1 and HEV-2 in neighboring Nigeria (PMID: 30352598). In our study, the absence of HEV-1 and HEV-2 detection in wastewater may be attributed to the limited sampling size and geographic coverage, which may not fully capture the diversity of circulating HEV genotypes. It is possible that HEV-1 and HEV-2 are present in the population but were not detected due to the restricted temporal and spatial scope of sampling. We have included this point in the discussion to acknowledge this limitation and recommend broader surveillance in future studies (**line 254-257)**.

λ Discussion, line 202: The sentence is overstated. The findings in this study does not support the hypothesis of zoonotic transmission of HEV in Cameroon, unless identical HEV sequences could be identified in both humans and animals. Please revise it.

Thank you for this important observation. We acknowledge that the initial sentence may have exaggerated the results. The sentence in **line 243-245** has been revised to reflect a more cautious interpretation of the data. While the study finds evidence of HEV in wastewater, it does not provide direct evidence of zoonotic transmission. “These cases suggest that this rodent-associated virus may be crossing into human populations more often than previously thought, and could be an emerging zoonotic threat.”

λ Notably, references 19, 22, and 23 are identical! Please ensure that the references are properly cited.

Thank you for the remark. The references have been revised and are now properly cited in the updated manuscript.

**Minor comments:**
λ Line 47: Humans are also hosts of HEV-3. Please refer to the relevant review (PMID: 32810801).

We agree with Reviewer #1’s comment that humans are also hosts of HEV genotype 3 (HEV-3). To address this, we have revised the manuscript and added the following sentence: “HEV genotype 3 (HEV-3) is zoonotic and widely distributed among both humans and animals, particularly in developed and transitional countries.” **Line 68 to 69.** In addition, we have included the following reference to support this statement: Wang B, Meng X-J. Structural and molecular biology of hepatitis E virus. Comput Struct Biotechnol J. 2021;19:1907–1916. doi:10.1016/j.csbj.2021.03.038

λ Line 79: This sentence is figure legend which should be removed in the main text.

Thank you for the remark. The sentence has been removed from the revised manuscript as suggested.

λ Line 92: Define “ORF”.

We thank the reviewer for the comment regarding the definition of ORF1. We have revised the manuscript and added the definition as follows on **line 124**:“ORF1 (Open Reading Frame 1)”

λ Line 93: The reference 2 is irrelevant.

We agree with Reviewer #1’s comment regarding reference 2. Accordingly, we have removed this reference from the corresponding line 115 in the manuscript.

λ Line 106: Change “readability” to “accuracy”.

Thank you for the suggestion. The change was made as proposed, and the reference has been removed from line 128 in the revised manuscript.

λ Lines 110 and 115: “hepatitis E virus” has already been defined as “HEV” earlier.

Thank you for the suggestion. As proposed, we have used only the acronym HEV in the revised manuscript, since it has already been defined earlier as Hepatitis E virus.

λ Lines 112-114: Please define the abbreviations.

Thank you for the remarks. We would like to clarify that CHUY refers to the University Hospital Center, and it is the only acronym used. The other names—Diderot, Mvog Ada, Bonas, Paposy, and Biyem Assi—are not abbreviations but rather the actual names of the sampling sites, And the words was lowercase.

λ Lines 124-125: Remove this sentence which is a Table title.

Thank you for the remark. The table has been added as proposed by the editor in the revised manuscript.

λ Line 137: Remove this sentence of figure legend.

Thank you for the remark. The sentence has been removed from the revised manuscript as suggested.

λ Line 140: It should be “Figure 3”.

Thank you for the remark. The sentence has been removed from the revised manuscript as suggested.

λ Lines 148-151: Remove the figure legend in the main text.

Thank you for the remark. The sentence has been removed from the revised manuscript as suggested.

λ Unify “HEV-3” and “HEV genotype 3” throughout the manuscript.

Thank you for the suggestion. We have unified the terminology/naming as requested in the revised manuscript.

λ Line 214: Delete “(2)”.

Thank you for the observation. We have removed the indicated content from the revised manuscript.

**Reviewer #2:** This study illustrates the interest of wastewater based epidemiology for HEV, an enteric virus that can be transmitted zoonotically. Despite the low number of water samples analysed this study indicates the predominance of HEV genotype 3 circulation in Yaounde, Cameroon, rather than HEV genotypes 1 or 2 that are strictly found in human. This topic is interesting but the manuscript needs corrections and can be improved.

**Comments**
1. Methods
The region targeted in ORF1 gene for PCR detection and sequencing must be indicated. What is the limit of detection of the assay? Wastewater can contain PCR inhibitors; how was the analytical process controlled?

To conform to the reviewer, we add these sentences in the methods section, **lines 115 to 119** as follows:

Briefly, 500 mL of wastewater was pre-treated by centrifugation at 1,940 g and 4 °C for 30 minutes to minimise the presence of PCR inhibitors. The resulting supernatant was then chemically treated using a 1 mol/L NaOH solution. Next, a mixture of 39.5 mL of 22% dextran, 287 mL of 29% PEG 6000, and 35 mL of 5 M NaCl solution was added. The resulting pellet was treated with chloroform to obtain a clear sample and Gentamicin 100 mg/mL reduce bacterial contamination. In addition lines 118 and 119 were also added as follows: Total DNA/RNA were extracted from 200 µL of concentrated wastewater using a commercial purification kit with an inhibitor removal spin column (DaAnGene®), following the manufacturer’s instructions.

2. Results, line 116 – 123 There are discrepancies between the text and table 1 : eg BONAS detection rate at 10.5% (2/19) line 116 and 41.7% (5/12) in Table 1; no corresponding site for 15.8% (3/19) line 116; repetitions lines 121-123.

We agree with Reviewer #2’s comment regarding the lack of coherence in the description of the results. The description has been revised and updated in the manuscript to improve clarity and consistency. **Line 152-156:** As shown in Table 1, HEV was detected in 26.4% (19 out of 72) of wastewater samples. Notably, HEV was identified in samples from all six study locations. The highest positivity rates were observed in Bonas and Paposy, at 41.7% (05 out of 12 samples each), while the lowest rate was recorded in CHUY at 8.4% (one out of 12 samples). Biyem Assi and Diderot showed positivity rates of 25% (03 out of 12 samples), while Mvog Ada's rate was 16.7% (two out of 12 samples).

3. Results line 126 : Is the signal intensity sufficiently reliable ? Was the signal intensity reproducible in duplicate PCR experiments ? Fig 2a is not very useful.

We thank the reviewer for these pertinent observations. In response, we have removed the term “strong” from the manuscript, as it may have implied a quantitative interpretation that was not rigorously supported **line 170** “The HEV signal detections were found at the plant watering point and in effluent from domestic sewage systems”). Signal intensity was assessed visually from the agarose gel electrophoresis results, which is inherently semi-quantitative and may lack reproducibility for comparing samples. To address the concern regarding reproducibility, we confirm that all positive PCR results were obtained in at least two independent PCR runs using the same RNA extracts, to ensure consistency and reduce the chance of false positives. Additionally, in light of the reviewer’s comment, we have removed Figure 2a from the manuscript, as its contribution to the overall analysis was limited.

4. The detection rate according to the different sites could be related to the population size. A potential relationship should be analysed.

We thank the reviewer for this insightful observation. We agree that population size and density at each sampling site may influence HEV detection rates, as higher population concentrations can result in increased viral shedding into wastewater. However, in the present study, we did not have access to reliable or disaggregated demographic data to perform a robust correlation analysis between population size and HEV detection. We have now acknowledged this limitation in the discussion section of the revised manuscript and emphasized the need for future studies to integrate population-based data. **Line 254-255:** “The sample size was relatively small, and half of the sampling sites were residential areas, which might not fully represent the city’s wastewater landscape”. Such information would enable a more accurate assessment of the relationship between community size, sanitation infrastructure, and viral circulation in wastewater.

5. Phylogenetic analysis: Reference HEV sequences (Smith et al J Gen Virol 2020) must be used for determining HEV-3 subtype. This is relevant because HEV subtype 3a was previously detected in Cameroon and a comparison with HEV 3 subtypes detected in animals could provide additional informations

We thank the reviewer for this valuable and relevant suggestion. In response, we updated our phylogenetic analysis by including the official HEV-3 subtype reference sequences as proposed by Smith et al., Journal of General Virology (2020), to allow a more accurate classification of our sequences and enable direct comparison with known HEV-3 subtypes, including those previously reported in Cameroon. After adding these reference sequences, we observed that our sequences did not cluster with any known HEV-3 subtype. Instead, they consistently formed a separate clade, positioned in close proximity to genotype C1 of Orthohepevirus C. This finding was supported by both neighbor-joining and maximum likelihood methods with strong bootstrap support. Although HEV-3a has been previously identified in human and animal samples in Cameroon, our wastewater-derived sequences appear genetically distinct from this group. These results strengthen our initial hypothesis that the HEV strains detected in this study may have a different origin, potentially linked to rodent-associated HEV-C1-like strains. We have updated Figure 3 accordingly and clarified these findings in both the results and discussion sections of the revised manuscript. We also emphasize in the discussion the need for further investigation, particularly whole-genome sequencing and comparative analysis with local animal HEV strains, to confirm this unexpected phylogenetic positioning. Figure 3. The update was added on discussion, conclusion of the manuscript section **line 234-283;** and in result and conclusion of abstract **line 40-51.**

6. Discussion: HEV RNA was detected in Yaounde wastewaters. The source is probably from humans infected with HEV but an animal source is also possible (eg infected pigs)? This point should be documented and discussed.

We thank the reviewer for this relevant observation. We acknowledge that urban wastewater can contain viral excretions from human sources, but also from animals, particularly in environments where contact between humans, domestic or wild animals, and poorly managed sanitation systems is frequent. While a human origin of the HEV detected in Yaoundé wastewater is the most likely explanation, an animal source especially pigs cannot be ruled out, given the documented presence of HEV-3 in pigs in Cameroon (refs. 20, 21). We have added a paragraph in the discussion to address this possibility. In the absence of direct data from animal samples in our study, we cannot currently determine the exact source of the virus with certainty. However, our findings particularly the phylogenetic proximity to genotype C1 of the Orthohepevirus C group highlight the need for comparative studies including samples from local pigs and rodents. Such an integrated approach, within a One Health framework, would help clarify the transmission pathways and potential reservoirs of HEV in Yaoundé. This point has been clarified in the revised version of the manuscript, specifically in the Discussion section and in the recommendations for future research. **Line 246-258**.

7. Discussion, lines 165-167: Beside foodborne transmission of HEV, waterborne transmission is a possibility. However, the detection of HEV in wastewater does not prove waterborne transmission because this reflects only excretion of the virus from infected individuals.

We thank the reviewer for this valuable observation. We agree that while the detection of HEV RNA in wastewater confirms viral excretion by infected individuals, it does not constitute definitive evidence of waterborne transmission. Accordingly, we have revised the text on **lines 203–207** as follows: “This observation highlights that the presence of HEV in the environment, resulting from viral excretion by infected individuals, may contribute to exposure risk in Cameroon. It suggests that untreated wastewater can serve as a useful tool for monitoring HEV and other enteric pathogens within the community, even during non-epidemic periods.

8. References: All the references must be checked. Duplicate and triplicate were detected (eg ref 16 and 30 ; ref 19, 22 and 23 with confusion between names and surnames)

We agree with Reviewer #2’s comment regarding the duplication of some references. We have corrected this issue in the revised manuscript.

9. Figures and Table :
- Fig 1 legend must give more detail
- Fig 3 is missing
- Table 1 : a column with the sampling area should be added.

We appreciate the reviewer’s helpful comments regarding the figures and table presentation.

Figure 1 legend: We have revised the legend for Figure 1 to include additional details that clarify the data presented and enhance the figure’s interpretability.

Figure 3: We apologize for the omission. Figure 3 has now been included in the revised manuscript.

Table 1: A new column indicating the sampling area has been added to Table 1, as suggested.

**10. Minor points in introduction**
- Line 41 : add contaminated food

We thank the reviewer for this suggestion. We have updated the sentence on **line 57** to include "contaminated food" as an additional transmission route.

- Line 47: five instead of four

We thank the reviewer for pointing this out. The sentence has been corrected to state “five” instead of “four” genotypes. The revised sentence now reads: Among the eight identified HEV genotypes (HEV-1 to HEV-8), **line 67** five (HEV-1, HEV-2, HEV-3, HEV-4 and HEV-7)

- Line 53 : HEV4 mainly found in Asia

We thank the reviewer for this observation. The sentence has been revised to reflect that HEV-4 is mainly found in Asia, with some circulation also reported in Europe. The updated sentence now reads: **line 77** HEV-4 is mainly found in Asia and Europe

- Line 55-56 : persistent infectious lead to liver cirrhosis (and not the reverse)

We thank the reviewer for highlighting the importance of clarifying the causal relationship. We agree that persistent HEV infection leads to liver cirrhosis, rather than cirrhosis causing persistent infection. Accordingly, we have revised the sentence to better reflect this causality **line 78-81**: In contrast, HEV-3 and HEV-4 are more prevalent in industrialized countries and can cause persistent infections in immunocompromised individuals, which may subsequently lead to accelerated progression toward liver cirrhosis.

- Line 63 : add reference 16

We thank the reviewer for this important comment. **Line 88** We have added reference [16] to support the statement regarding the detection of HEV in wastewater samples from several countries. The revised sentence now reads: The presence of HEV in drinking and irrigation water is a growing public health concern, as it can contaminate these vital resources. HEV has been detected in wastewater samples from several countries [16]
